# Skin cancer awareness, attitude, and sun protection behavior among medical students in Saudi Arabia: A cross-sectional study

**DOI:** 10.1097/MD.0000000000044913

**Published:** 2025-10-24

**Authors:** Salman Bin Dayel

**Affiliations:** a Dermatology Unit, Department of Medicine, College of Medicine, Prince Sattam Bin Abdulaziz University, Al-Kharj, Saudi Arabia.

**Keywords:** skin cancer, sunlight exposure, sunlight protection, sunscreen, UV indexing

## Abstract

Skin cancer is a significant public health concern, and awareness of the risks associated with ultraviolet exposure and the adoption of protective measures are essential for prevention. This study aimed to assess the level of awareness, attitudes, and behaviors related to sun exposure risk and protection measures among medical students in Saudi Arabia. A cross-sectional study was conducted among 659 medical students from 22 different universities, primarily from the central region of Saudi Arabia. Participants completed a self-constructed questionnaire comprising personal questions, closed-ended questions about sunscreen knowledge and usage, awareness of sun exposure’s harmful effects on the skin, and factors predisposing to skin cancer. Additional questions assessed participants’ attitudes towards sun protection. Approximately 53.72% of the students reported not regularly using sunscreen, while the majority (76%) used sunglasses and made efforts to avoid sun exposure (87%). However, there remains a need to increase awareness regarding the risks of sun exposure leading to skin cancer. Significant differences were observed in attitudes and behaviors (*P* = .001), with females displaying greater awareness than males, as reflected in various criteria. Notably, 40% of females reported using sunscreen, compared to only 6% of males. Although awareness and behavior increased with academic level, these differences remained statistically insignificant. The study highlights the need for enhanced awareness programs and skin cancer campaigns, particularly among male medical students. Despite some positive attitudes and behaviors observed, there is still room for improvement in promoting sun protection measures among this population, contributing to primary prevention efforts against skin cancer.

## 1. Introduction

Solar radiation is the main source of ultraviolet radiation (UVR). Sun exposure becomes a main factor for the main 3 skin cancer types, squamous cell carcinoma basal cell carcinoma, and melanoma.^[1]^ The linkage between skin cancer and sun exposure was made by around 90% of study participants in Australia, 85% in Canada, 92% in the United States, and 92.5% in Malta.^[2]^ The prevalence of skin cancer has increased during the past few decades, and it accounts for 1 in 3 cancer cases worldwide, Behavioral interventions are needed,^[3]^ and avoidance of ultraviolet (UV) radiation has the potential to reduce the risk of skin cancer.^[4]^

Nonmelanoma skin cancer is the most common malignancy in the United States with an estimated 0.9 to 1.2 million new cases each year.^[5]^ Approximately 3 million nonmelanoma skin cancers and 1,32,000 new cases of melanomas are diagnosed globally each year.^[6]^ Every day people are extensively exposed to UVR, especially in the area of high UV indexing, and also through several other sources such as: living and traveling in sunny climates, excessive sunbathing and sunbed use, outdoor sports, and usage of appliances and devices that emit UVR in domestic and industrial settings.^[7–9]^

Protective measures and being aware of skin cancer were shown to be effective in avoiding the harmful effects of UVR and thereby preventing skin cancer.^[8,10]^ Sunscreen has proven to have a major role in preventing skin cancer.^[11]^ Sunscreens work as a barrier to protect against UVR by blocking the radiation entrance through the skin. In Saudi Arabia, there are yet no population-based studies measuring the prevalence of sunscreen use.^[12]^ Very few studies have been done to examine the importance of sunscreen,^[11]^ As they are future physicians, the knowledge and awareness of medical students about skin cancers and the importance of sunscreen use will have an impact on the population and they will play an important role in primary prevention. It represents a key component of skin cancer prevention.^[13,14]^

We intended our study to test the awareness of the Saudi medical students of skin cancer and the risk of sun exposure. We also want to test the attitude and behavior toward sun protective measures.

## 2. Materials and methods

This cross-sectional study was conducted between March and April 2022 to assess sun protection awareness and behaviors among medical students at 22 universities across Saudi Arabia. The estimated target population consisted of approximately 16,500 students.

The sample size was calculated using OpenEpi (2016), which estimated that a sample of 638 participants would provide a 99% confidence level. Ultimately, 775 students received the survey, of whom 659 completed it (85% response rate), exceeding the required sample size.

Participant recruitment was coordinated through university student committees and class representatives, using both printed handouts and electronic links distributed via institutional emails and student groups. Printed questionnaires were completed by 560 students, and 215 responded to the online version.

The self-constructed questionnaire comprised 3 main sections. The first included 5 demographic questions (sex, age, university, academic year, and city). The second featured 12 closed-ended items on participants’ knowledge regarding sunscreen, harmful effects of sun exposure, and skin cancer risk factors. The third section included 7 closed-ended items evaluating attitudes toward sun protection practices.

To ensure reliability and clarity, the questionnaire was reviewed by a panel of 3 dermatology experts and underwent pilot testing with 30 participants. Based on the pilot results, minor revisions were made. Internal consistency was assessed using Cronbach’s alpha (α = 0.79), indicating acceptable reliability.

The final version of the survey was anonymized and self-administered, either on paper or through an online form. Informed consent was obtained from all participants. This study was based on anonymous, voluntary survey responses from medical students, and it was determined by the Institutional Review Board at Prince Sattam bin Abdulaziz University that ethical approval was not necessary for this type of observational study.

## 3. Results

A total of 659 medical students from 22 universities across Saudi Arabia participated in the study. The majority of participants were from the central region, accounting for 89.36% of the total sample (Table 1).

**Table 1:** The table shows the demographic characteristics.

Gender	
** **Male	279 (42.34%)
** **Female	380 (57.66%)
Age group	
** **18–20	261 (39.61%)
** **21–23	322 (48.86%)
** **24–26	71 (10.77%)
** **More than 26	5 (0.76%)
Academic year	
** **First	127 (19.27%)
** **Second	128 (19.42%)
** **Third	173 (26.25%)
** **Fourth	114 (17.3%)
** **Fifth	106 (16.08%)
** **Intern	11 (1.67%)
Region	
** **Central region	588 (89.36%)
** **Eastern region	12 (1.82%)
** **Western region	47 (7.14%)
** **Northern region	8 (1.22%)
** **Southern region	3 (0.46%)

Regarding sun protection behavior, the data revealed that 53.72% of the students reported not regularly using sunscreen, even when going to the beach. Additionally, 43.4% stated they do not use sunscreen at all. Only 12.75% of participants reported using tanning booths, while a substantial proportion (87%) indicated that they try to avoid sun exposure when possible. The majority of students (76%) reported using sunglasses as a form of protection (Table 2).

**Table 2:** The table shows the behavior of the students toward the protective tools.

	No	Sometimes	Yes
Do you use sunscreen	354 (53.72%)	159 (24.13%)	146 (22.15%)
Do you use sunscreen when you go out to the swimming pool or the beach?	286 (43.4%)	148 (22.46%)	225 (34.14%)
Do you do tanning?	484 (73.44%)	91 (13.81%)	84 (12.75%)
Do you try to sit in the shadows to avoid the sun exposure?	83 (12.59%)	201 (30.5%)	375 (56.9%)
Do you try to avoid sun exposure from 10 am to 4 pm?	339 (51.44%)	65 (9.86%)	255 (38.69%)
Do you wear sunglasses?	215 (32.63%)	175 (26.56%)	269 (40.82%)

In terms of awareness and knowledge, 16.69% of respondents were unaware of the link between sun exposure and skin cancer, and 20.33% believed that sun exposure carries no risk of skin cancer. Awareness of geographic location as a risk factor was reported by 64%, yet only 22.9% acknowledged personal predisposition to skin cancer. Misconceptions were also observed regarding hereditary factors; 17.9% believed there is no hereditary role, while 27% were unsure. Additionally, only 18.82% were aware that individuals with darker skin are less susceptible to sun-induced skin cancer (Table 3).

**Table 3:** The table demonstrates the awareness of medical students about skin cancer and its predisposing factors.

	I don’t know	No	Yes
Dark-skin people don’t need sun protection	188 (28.53%)	347 (52.66%)	124 (18.82%)
You are predisposed to skin cancer	249 (37.78%)	259 (39.3%)	151 (22.91%)
Sun exposure is a major risk factor for skin cancer	110 (16.69%)	134 (20.33%)	415 (62.97%)
The geographic location is a factor of skin cancer	134 (20.33%)	95 (14.42%)	430 (65.25%)
Hereditary has a factor of skin cancer	178 (27.01%)	118 (17.91%)	363 (55.08%)

The majority of students (73%) recognized the importance of sunscreen use. Among them, 38% knew it should be applied at least 20 minutes before sun exposure, 32.5% were aware of the need to reapply it after 90 minutes of exposure, and 36.12% believed it should also be applied when inside a car. However, only 22.8% reported using sunscreen on cloudy days. Despite understanding its benefits, 31.26% cited cost as a barrier to regular use, and 28.22% reported lack of time as a deterrent (Table 4).

**Table 4:** The table demonstrates the attitude of the medical students toward the sunscreen importance and the usage.

	I don’t know	No	Yes
Sunscreen is important	115 (17.45%)	63 (9.56%)	481 (72.99%)
Sunscreen is expensive	286 (43.4%)	167 (25.34%)	206 (31.26%)
Sunscreen should be repeated after 90 min of sun exposure	284 (43.1%)	161 (24.43%)	214 (32.47%)
It’s better to put sunscreen 20 min before you leave the house	293 (44.46%)	115 (17.45%)	251 (38.09%)
You don’t have time to use sunscreen	301 (45.68%)	172 (26.1%)	186 (28.22%)
It’s important to use sunscreen while you are in the car	197 (29.89%)	224 (33.99%)	238 (36.12%)
It’s important to use sunscreen in cloudy day	168 (25.49%)	343 (52.05%)	148 (22.46%)
It’s important to use sunscreen even if you will be exposed to sun for less than 1 h	214 (32.47%)	198 (30.05%)	247 (37.48%)

Chi-square and Fisher’s exact tests were used to examine gender-based differences in awareness and behaviors (SPSS v26; SPSS Inc., IBM, Chicago). Missing data were handled using listwise deletion.

**Figure 1.**
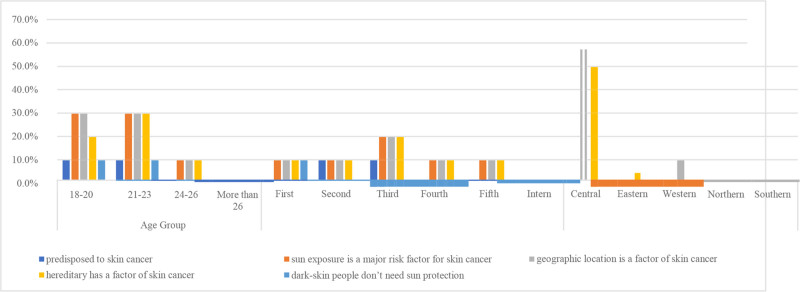
The figure shows the comparison in attitude between male and female students.

A gender-stratified analysis revealed notable disparities. Female students were generally more informed and proactive about sun protection. For example, approximately 40% of females used sunscreen compared to only 6% of males. Female students were also more likely to avoid direct sunlight, seek shade, and wear sunglasses. Interestingly, a relatively high proportion of female students (21%) reported using tanning booths. While increased awareness and protective behavior were observed across academic levels, these differences were statistically insignificant (Table 5, Fig. 1).

**Table 5:** The table shows the comparison in the attitude between the males and females skin cancer.

	Gender		*P* value
	Male	Female	
Do you think dark-skin people don’t need sun protection?			
I don’t know	96 (14.57%)	92 (13.96%)	
No	125 (18.97%)	222 (33.69%)	.002
Yes	58 (8.8%)	66 (10.02%)	
Do you think you are predisposed to skin cancer?			
I don’t know	114 (17.3%)	135 (20.49%)	
No	97 (14.72%)	162 (24.58%)	.123
Yes	68 (10.32%)	83 (12.59%)	
Do you think sun exposure is a major risk factor for skin cancer?			
I don’t know	58 (8.8%)	52 (7.89%)	
No	69 (10.47%)	65 (9.86%)	.001
Yes	152 (23.07%)	263 (39.91%)	
Do you think the geographic location is a factor of skin cancer?			
I don’t know	61 (9.26%)	73 (11.08%)	
No	32 (4.86%)	63 (9.56%)	.164
Yes	186 (28.22%)	244 (37.03%)	
Do you think hereditary has a factor of skin cancer?			
I don’t know	71 (10.77%)	107 (16.24%)	
No	41 (6.22%)	77 (11.68%)	.073
Yes	167 (25.34%)	196 (29.74%)	

**Figure 2.**
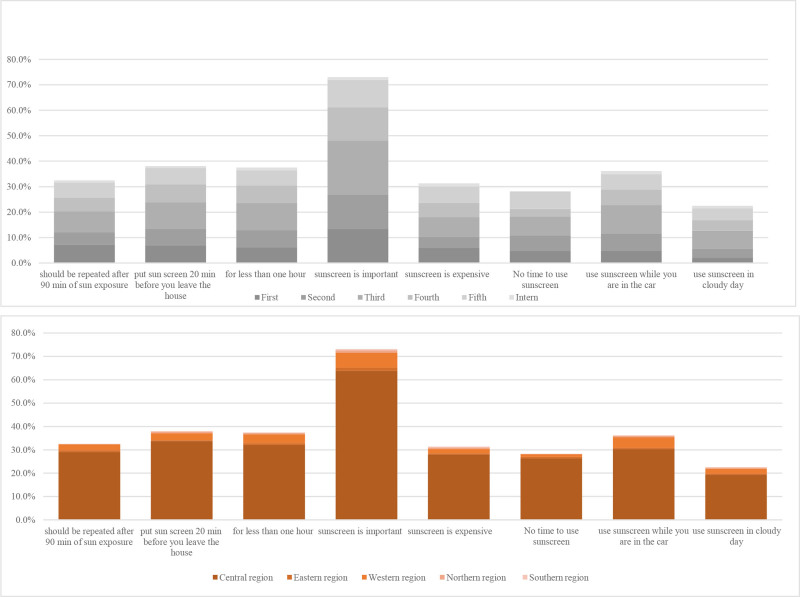
The figure shows the significance between males and females usage of protection measures.

Table 6 and Figure 2 summarize gender differences in sun protection practices. Statistically significant differences were observed in knowledge and behavior between male and female students (*P* < .05), particularly regarding sunscreen use and sun avoidance practices. Female students demonstrated superior knowledge about protection measures and a stronger awareness of the risks of unprotected sun exposure.

**Table 6:** The table shows the comparison in the attitude between the males and females to the sunscreen as a protection measurement.

	Gender		*P* value
	Male	Female	
Do you think sunscreen should be repeated after 90 min of sun exposure?			
I don’t know	153 (23.22%)	131 (19.88%)	.000
No	68 (10.32%)	93 (14.11%)	
Yes	58 (8.8%)	156 (23.67%)	
Do you think it’s better to put sun screen 20 min before you leave the house?			
I don’t know	137 (20.79%)	156 (23.67%)	.000
No	62 (9.41%)	53 (8.04%)	
Yes	80 (12.14%)	171 (25.95%)	
Do you think sunscreen is important?			
I don’t know	79 (11.99%)	36 (5.46%)	.000
No	40 (6.07%)	23 (3.49%)	
Yes	160 (24.28%)	321 (48.71%)	
Do you think sunscreen is expensive?			
I don’t know	172 (26.1%)	114 (17.3%)	.000
No	49 (7.44%)	118 (17.91%)	
Yes	58 (8.8%)	148 (22.46%)	
Do you think you don’t have time to use sunscreen?			
I don’t know	136 (20.64%)	165 (25.04%)	.000
No	44 (6.68%)	128 (19.42%)	
Yes	99 (15.02%)	87 (13.2%)	
Do you think it’s important to use sunscreen while you are in the car?			
I don’t know	107 (16.24%)	90 (13.66%)	.000
No	121 (18.36%)	103 (15.63%)	
Yes	51 (7.74%)	187 (28.38%)	
Do you think it’s important to use sunscreen in cloudy day?			
I don’t know	74 (11.23%)	94 (14.26%)	.000
No	186 (28.22%)	157 (23.82%)	
Yes	19 (2.88%)	129 (19.58%)	
Do you think it’s important to use sunscreen even if you will be exposed to sun for less than 1 h?			
I don’t know	110 (16.69%)	104 (15.78%)	.000
No	103 (15.63%)	95 (14.42%)	
Yes	66 (10.02%)	181 (27.47%)	
Do you use sunscreen			
No	238 (36.12%)	116 (17.6%)	
Sometimes	30 (4.55%)	129 (19.58%)	.000
Yes	11 (1.67%)	135 (20.49%)	
Do you use sunscreen when you go out to swimming pool or the beach?			
No	200 (30.35%)	86 (13.05%)	
Sometimes	47 (7.13%)	101 (15.33%)	.000
Yes	32 (4.86%)	193 (29.29%)	
Do you do tanning?			
No	245 (37.18%)	239 (36.27%)	
Sometimes	21 (3.19%)	70 (10.62%)	.000
Yes	13 (1.97%)	71 (10.77%)	
Do you try to set in the shadow to avoid the sun exposure?			
No	26 (3.95%)	57 (8.65%)	
Sometimes	76 (11.53%)	125 (18.97%)	.009
Yes	177 (26.86%)	198 (30.05%)	
Do you try to avoid sun exposure from 10 am to 4 pm?			
No	126 (19.12%)	213 (32.32%)	
Sometimes	34 (5.16%)	31 (4.7%)	.016
Yes	119 (18.06%)	136 (20.64%)	
Do you use sunglasses?			
No	98 (14.87%)	117 (17.75%)	
Sometimes	59 (8.95%)	116 (17.6%)	.027
Yes	122 (18.51%)	147 (22.31%)	

## 4. Discussion

Exposure to UV radiation remains a well-established environmental risk factor in the development of skin cancer. Extensive epidemiological studies have identified UV exposure as a major contributor to squamous cell carcinoma, basal cell carcinoma, and melanoma.^[15,16]^ The carcinogenic effect of UV radiation is largely mediated through the induction of deoxyribonucleic acid mutations, particularly pyrimidine dimers, which can lead to genetic instability and malignant transformation.^[17]^

This study contributes meaningful insight into sun exposure awareness and protective behaviors among Saudi medical students – a demographic poised to influence public health education in the future. Despite being situated in a high UV index region, Saudi Arabia paradoxically reports lower skin cancer incidence compared to regions with similar exposure. This may be attributed to genetic factors, cultural dress habits, or differences in behavioral exposure, as highlighted in prior studies.^[18,19]^

One of the central findings of this study is the suboptimal use of sunscreen among participants. Over half (53.72%) reported not using sunscreen regularly, a concerning figure given the well-documented protective role of sunscreen in reducing UV-related skin damage and skin cancer risk.^[20]^ While debates persist regarding sunscreen efficacy – often attributed to factors like incorrect application or insufficient reapplication – evidence supports its utility when used appropriately.^[13,21]^ Therefore, we recommend intensified educational campaigns promoting correct sunscreen use, especially targeted toward populations at risk.

Encouragingly, a high proportion of students reported using sunglasses (76%) and actively avoiding direct sunlight (87%), behaviors aligned with global sun protection recommendations.^[22]^ However, significant knowledge gaps persist. Nearly 17% of respondents were unaware of the link between sun exposure and skin cancer, and over 20% believed there was no such risk. Furthermore, 37.78% were unaware of their personal susceptibility, and 27.01% were uninformed about hereditary risk – underscoring a pressing need for enhanced public health messaging and academic curriculum integration.^[23,24]^

Gender differences in sun protection practices were striking. Female students demonstrated significantly greater knowledge and engagement in protective behaviors, including sunscreen use (40% of females vs 6% of males). These results are consistent with prior literature highlighting gender-based disparities in health behaviors.^[20,25]^ This finding underlines the need for tailored awareness campaigns aimed specifically at male students, who remain less engaged despite equivalent risk.

A particularly concerning observation was that 12.75% of students reported using tanning booths. Indoor tanning is strongly associated with increased melanoma risk and should be actively discouraged.^[25]^ Campaigns should explicitly address the dangers of indoor tanning, especially among young adults, where cosmetic motivations often override health considerations.

Based on the study’s results, several key implications and recommendations can be drawn:

1. Expand education initiatives across medical colleges in Saudi Arabia focusing on UV risks, correct sunscreen use, and the role of protective clothing and behaviors.
2. Develop gender-sensitive interventions that specifically engage male students, who exhibit lower sun protection awareness and practices.
3. Emphasize genetic and personal risk profiling in educational content to improve understanding of individual susceptibility to skin cancer.
4. Discourage indoor tanning practices by informing students about the serious health risks associated with tanning booths.
5. Promote routine sunscreen use, particularly emphasizing its importance even on cloudy days or while indoors near UV-exposed windows.
6. Enhance curricular training for medical students on skin cancer prevention strategies to empower them as future health educators.

## 5. Limitations and future directions

This study had several limitations that may impact the generalizability of findings. The participant pool was limited to medical students and predominantly drawn from the central region of Saudi Arabia, which may not reflect national patterns. Additionally, the reliance on a self-reported, self-constructed questionnaire introduces potential for reporting and measurement bias. While the questionnaire underwent expert validation and pilot testing (Cronbach’s α = 0.79), future studies should utilize standardized tools validated across diverse populations. Moreover, this study employed univariate analysis, which restricts our ability to control for confounding variables.

In future research, employing multivariable analysis and including broader demographic representation will enhance the robustness and generalizability of findings.

## 6. Conclusion

The results of this study underscore the importance of enhancing skin cancer awareness and sun protection behaviors among medical students in Saudi Arabia. Despite some positive attitudes and behaviors observed, there remains room for improvement in promoting sun protection measures among this population. The findings provide a roadmap for developing targeted interventions and educational campaigns to reduce the incidence of skin cancer in the region and improve overall public health.

## Acknowledgments

This study is supported via funding from Prince Sattam bin Abdulaziz University project number (PSAU/2023/R/1444).

## Author contributions

**Conceptualization:** Salman Bin Dayel.

**Data curation:** Salman Bin Dayel.

**Formal analysis:** Salman Bin Dayel.

**Funding acquisition:** Salman Bin Dayel.

**Investigation:** Salman Bin Dayel.

**Methodology:** Salman Bin Dayel.

**Project administration:** Salman Bin Dayel.

**Resources:** Salman Bin Dayel.

**Software:** Salman Bin Dayel.

**Supervision:** Salman Bin Dayel.

**Validation:** Salman Bin Dayel.

**Visualization:** Salman Bin Dayel.

**Writing – original draft:** Salman Bin Dayel.

**Writing – review & editing:** Salman Bin Dayel.
